# SURWEY real-world study of solriamfetol: initiation, titration, safety, efficacy, and follow-up experience for patients with obstructive sleep apnea in Germany

**DOI:** 10.1007/s11325-025-03275-6

**Published:** 2025-02-27

**Authors:** Yaroslav Winter, Geert Mayer, Heike Benes, Lothar Burghaus, Samantha Floam, Gregory S. Parks, Ulf Kallweit

**Affiliations:** 1 Department of Neurology, University of Saarland, Homburg, Germany; 2 Department of Neurology, Philipps University Marburg, Marburg, Germany; 3 Hephata Klinik, Schwalmstadt, Germany; 4 Somni bene GmbH Institut für Medizinische Forschung und Schlafmedizin Schwerin GmbH, Schwerin, Germany; 5 Department of Neurology, Heilig Geist-Hospital, Cologne, Germany; 6 Axsome Therapeutics, New York, USA; 7 Formely of Axsome Therapeutics, New York, USA; 8 Center for Biomedical Education and Research, and Professorship for Narcolepsy and Hypersomnolence Research, Department of Medicine, University Witten/Herdecke, Witten, Germany; 9 Department of Neurology, University of Saarland, Homburg, Germany Kirrberger Str, 66421

**Keywords:** Obstructive sleep apnea, Excessive daytime sleepiness, Europe, Germany, Solriamfetol, Real-world evidence

## Abstract

**Purpose:**

Solriamfetol is approved for use in the European Union to treat excessive daytime sleepiness (EDS) associated with obstructive sleep apnea (OSA). SURWEY characterized real-world evidence regarding physician initiation and titration strategies and patient experiences with solriamfetol. We report SURWEY data for patients with OSA and EDS in Germany (*N* = 83).

**Methods:**

SURWEY was a retrospective chart review conducted among physicians in Germany. Eligible patients were age ≥ 18 years who reached a stable solriamfetol dose and completed ≥ 6 weeks of treatment. Patients were grouped by solriamfetol initiation strategy: changeover, add-on, new-to-therapy.

**Results:**

Patients’ mean (SD) age was 49 (14) years. New-to-therapy was the most common initiation strategy. Solriamfetol was initiated at 37.5 mg/day in most patients (*n* = 57, 69%) and titrated in 53 patients (64%); 30 (57%) completed titration within 2 weeks. In a post-hoc analysis, mean (SD) Epworth Sleepiness Scale (ESS) score was 16.0 (3.2) at baseline and decreased by 5.4 (3.6) at final follow-up (~ 16 weeks; *p* <.001). Improvement in patient- and physician-rated EDS was reported by ~ 90% of patients. Most patients (55%) reported effects of solriamfetol lasting ≥ 8 h; 91% of patients reported no change in nighttime sleep quality. The most frequent adverse events were headache (8%), decreased appetite (7%), and insomnia (6%).

**Conclusion:**

Most patients in this study were new to therapy. Solriamfetol was typically initiated at 37.5 mg/day; titration was common. ESS scores improved with solriamfetol treatment, and most patients self-reported improvement in EDS symptoms. Common adverse events were consistent with those reported in previous clinical trials.

## Introduction

Obstructive sleep apnea (OSA) is characterized by repeated obstruction of the upper airway following intermittent collapse of the pharynx during sleep, leading to episodes of sleep fragmentation and hypoxia [1]. Excessive daytime sleepiness (EDS), fatigue, and impaired cognitive functioning are often reported in patients with OSA [2]. Positive airway pressure (PAP) therapy is the first-line treatment to improve sleep in patients with OSA; however, EDS can persist in patients despite the use of PAP or other OSA therapies, even when CPAP use has been optimized and comorbid sleep disorders have been excluded, which may pose challenges in the clinical management of symptoms [2–6]. Patients with EDS associated with OSA can incur impaired occupational and social functioning, increased risk of workplace injuries and motor vehicle accidents, and reduced quality of life [1, 7, 8].

Solriamfetol (Sunosi^®^), a dopamine and norepinephrine reuptake inhibitor [9] with agonistic effects at the trace amine–associated receptor 1 and serotonin 1 A receptors [10, 11], is approved for use in adults in the European Union, Canada and the United States for the treatment of EDS associated with OSA (37.5–150 mg/day) and narcolepsy (75–150 mg/day) [12, 13]. The efficacy and safety of solriamfetol in patients with EDS associated with OSA have been demonstrated in short- and long-term clinical trials [14, 15]. The safety profile of solriamfetol is consistent across studies, in which common adverse events included headache, nausea, decreased appetite, diarrhea, dry mouth, insomnia, and anxiety [14, 15].

Although clinical studies have demonstrated the benefits of solriamfetol in the treatment of OSA-associated EDS, there is limited real-world data regarding physicians’ prescription and treatment initiation strategies and their outcomes. Such data may help healthcare providers optimize patient care in a clinical setting. Findings regarding real-world solriamfetol titration and administration strategies in US populations with EDS associated with OSA or narcolepsy have been reported [16], but no data is available on real-world outcomes in patients with EDS in OSA. The SUnosi Real World Experience studY (SURWEY) was designed to characterize the dosing and titration strategies used by European physicians initiating solriamfetol, as well as the patient outcomes following initiation. Data from a cohort of patients with narcolepsy in Germany have been described [17]. Here, we report additional SURWEY data for a cohort of patients with EDS associated with OSA in Germany.

## Materials and methods

### Study design

The SURWEY retrospective chart review study, conducted among physicians in Germany, enrolled “sleep clinic” or “other” sites that had physicians who were experienced in prescribing solriamfetol and were treating patients with EDS associated with OSA and/or narcolepsy [17]. This study, open from February 2021 through March 2023, was conducted in accordance with applicable national and local requirements for good research study practices. Required country-specific documentation was reviewed and approved, per local regulations, before any patient chart data were included in the study. Because all patient chart data were de-identified and anonymous to the sponsor, informed consent was not required from patients, based on international regulations, including the General Data Protection Regulation (GDPR).

### Data source and participants

The present analysis includes data from a cohort of patients with OSA and associated EDS treated with solriamfetol in Germany. Only physicians experienced in the diagnosis and management of OSA, who had prescribed solriamfetol for ≥ 10 patients before the end of the recruitment period, were eligible to participate. Consenting physicians were given detailed instructions and were trained to identify and select the patient charts that met the study’s eligibility criteria. These physicians identified patient charts and provided relevant information via electronic case report forms—a process expected to take ~ 30 min per chart. Additional information was provided through patient self-report. Eligible patients were ≥ 18 years of age, had a diagnosis of EDS associated with OSA, had reached a stable maintenance dose of solriamfetol, and completed ≥ 6 weeks of treatment; patients who received solriamfetol during a clinical trial or an early access program were excluded. To ensure a broad study population representative of patients with OSA and associated EDS, records from patients diagnosed with comorbidities, including psychiatric comorbidities, were included. The eligible patients were categorized into 3 subgroups, defined as follows: *changeover*, switched/switching from existing EDS medication(s) to solriamfetol; *add-on*, added solriamfetol to current EDS medication(s); and *new-to-therapy*, no current EDS medication at solriamfetol initiation.

### Endpoints and data analysis

The primary endpoint was the prescribed solriamfetol initiation dose and titration schedule, if applicable. Additional endpoints included patient type (changeover, add-on, new-to-therapy); change in ESS score from treatment initiation to final follow-up; physician and patient perceptions of improvement in EDS symptoms (“Impression of condition since initiation” rated on a Likert scale with points labeled *strongly improved*,* slightly improved*,* no change*,* slightly worsened*,* strongly worsened*, and *unknown*); perceived duration of effects of solriamfetol; change in nighttime sleep quality; and incidence of side effects. Data were analyzed for the safety population, defined as all patients with eligible charts; observed data are reported. The statistical evaluation was performed with SAS software, version 9.4 or higher. Data were summarized descriptively—continuous variables with n, mean, and SD statistics, and categorical variables with frequency counts and percentages. Post-hoc inferential analysis was conducted on change from baseline in ESS score using a paired samples t-test and on timing of solriamfetol wearing off with a one-sample Chi-squared goodness-of-fit test. Nominal p values are presented.

## Results

### Demographics of patients in Germany

This analysis included data from a total of 83 patients with OSA and associated EDS treated with solriamfetol. Mean (SD) age was 49 (14) years, 65% were male, and mean (SD) body mass index (BMI) was 32.2 (6.0) kg/m^2^ (Table 1). Of 76 patients who reported at least 1 comorbidity, the most common comorbidities included Obesity (58%), hypertension (49%) and anxiety/depression (30%). Overall, 73 patients (88%) used PAP therapy; 60 (72%) and 66 (80%) used PAP ≥ 4 h/night and ≥ 4 nights/week, respectively. New-to-therapy was the most common initiation strategy (*n* = 62, 75%), followed by add-on (*n* = 12, 14%) and changeover (*n* = 9, 11%). At baseline, mean (SD) ESS scores were 16.0 (3.2) overall and 16.6 (2.0), 16.3 (3.8), and 15.9 (3.2) in the changeover, add-on, and new-to-therapy subgroups, respectively.

**Table 1:** Baseline demographic and clinical characteristics

Characteristic	Changeover (*n* = 9)	Add-On (*n* = 12)	New-to-Therapy (*n* = 62)	Overall (*N* = 83)
Age, years, mean (SD)	44 (10)	40 (12)	51 (14)	49 (14)
Male, n (%)	7 (78)	7 (58)	40 (65)	54 (65)
BMI, kg/m^2^, mean (SD)	33.5 (4.0)	30.7 (5.8)	32.3 (6.2)	32.2 (6.0)
ESS score, mean (SD)	16.6 (2.0)	16.3 (3.8)	15.9 (3.2)	16.0 (3.2)
Any comorbidity, n (%)	9 (100)	11 (92)	56 (90)	76 (92)
Obesity	7 (78)	6 (50)	31 (50)	44 (53)
Hypertension	4 (44)	4 (33)	29 (47)	37 (45)
Anxiety/depression	2 (22)	4 (33)	17 (27)	23 (28)
Diabetes type 2	3 (33)	2 (17)	15 (24)	20 (24)
Hyperlipidemia	4 (44)	3 (25)	8 (13)	18 (22)
Migraine headache	1 (11)	2 (17)	9 (15)	12 (14)
Congestive heart failure	0	0	8 (13)	8 (10)
Coronary artery disease	1 (11)	1 (8)	5 (8)	7 (8)
Arrhythmia	0	0	6 (10)	6 (7)
Fibromyalgia	0	1 (8)	4 (6)	5 (6)
ADHD	2 (22)	0	0	2 (2)
Other	3 (33)	5 (42)	28 (45)	36 (43)

ADHD, attention-deficit/hyperactivity disorder; BMI, body mass index; ESS, Epworth Sleepiness Scale; SD, standard deviation

### Prior and concomitant medications for EDS

Overall, 30 patients reported prior use of medications for EDS (those taken *any* time before solriamfetol initiation; patients may have taken ≥ 1 of the medications listed), including 9 patients (75%) in the add-on subgroup, 9 patients (100%) in the changeover subgroup, and 12 patients (19%) in the new-to-therapy subgroup. The most common prior medications were pitolisant (*n* = 21, 70%), reported in 7/9 (78%), 8/9 (89%), and 6/12 patients (50%) in the add-on, changeover, and new-to-therapy subgroups who reported prior use of EDS medications, respectively, and modafinil (*n* = 12, 40%), reported in 2/9 (22%), 3/9 (33%), and 7/12 patients (58%) in the add-on, changeover, and new-to-therapy subgroups, respectively. Four patients (13%) reported use of other (nonspecified) medications (changeover, *n* = 1; new-to-therapy, *n* = 3).

Patients in the changeover group switched to solriamfetol from pitolisant (6 [67%]) or modafinil (1 [11%]); the switch from medication could not be determined in 2 patients (22%).

At the time of solriamfetol initiation, patients in the add-on group were taking pitolisant (7 [58%]), modafinil (2 [17%]) and other (nonspecified) medications (3 [25%]), and patients in the changeover group were taking modafinil (1 [11%]), pitolisant (2 [22%]), and other (nonspecified) medications (1 [11%]).

### Factors considered for solriamfetol initiation

When deciding to initiate solriamfetol treatment, the most commonly considered factor was patient comorbidities (*n* = 29, 35%), followed by prior medications (*n* = 20, 24%) and comedications (*n* = 11, 13%); physicians reported no specific influencing criteria for 45 patients (54%). Age, sex, and BMI were also cited as factors considered when initiating solriamfetol.

All 9 patients in the changeover group switched to solriamfetol because of lack of efficacy of prior medications; changeover was managed with an abrupt switch to solriamfetol from patients’ prior medications.

### Initiating and titrating solriamfetol

**Fig. 1:**
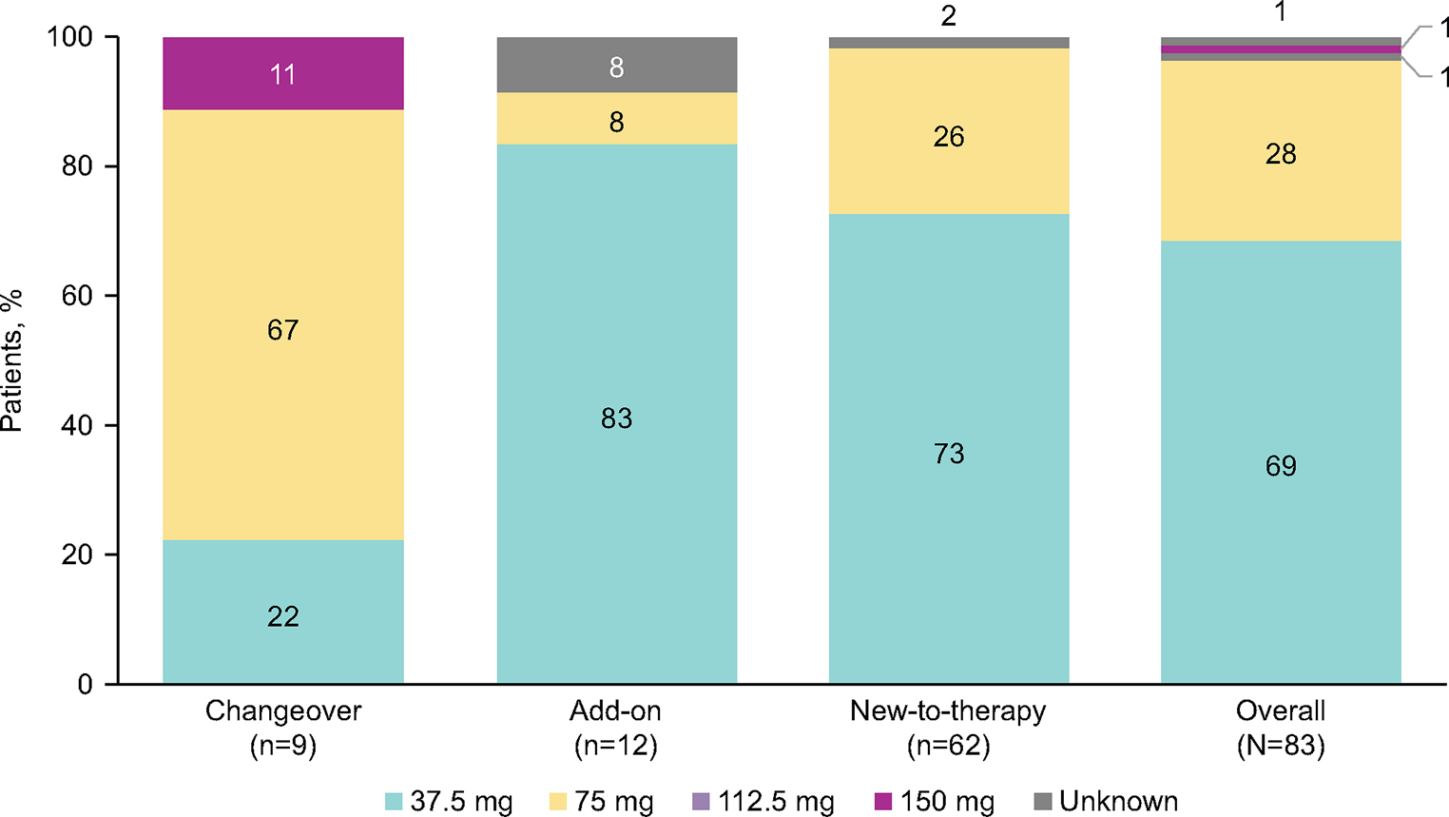
Starting Doses of Solriamfetol

98% of solriamfetol prescriptions were written for once-daily administration. The most common starting dosage of solriamfetol was 37.5 mg/day, followed by 75 mg/day (Fig. 1). Solriamfetol was titrated in 54 patients (65%), most of whom (*n* = 30, 56%) completed titration within 2 weeks; of those titrated, 48% (*n* = 26) completed titration within 7 days. All patients completed titration as prescribed.

### Change in ESS scores

Mean (SD) time from solriamfetol initiation to final follow-up was 15.6 (6.6) weeks overall (*n* = 82), which generally was consistent across therapy subgroups: changeover (*n* = 9), 13.8 (6.0) weeks; add-on (*n* = 12), 14.7 (5.9) weeks; new-to-therapy (*n* = 61), 16.0 (6.8) weeks.

**Fig. 2:**
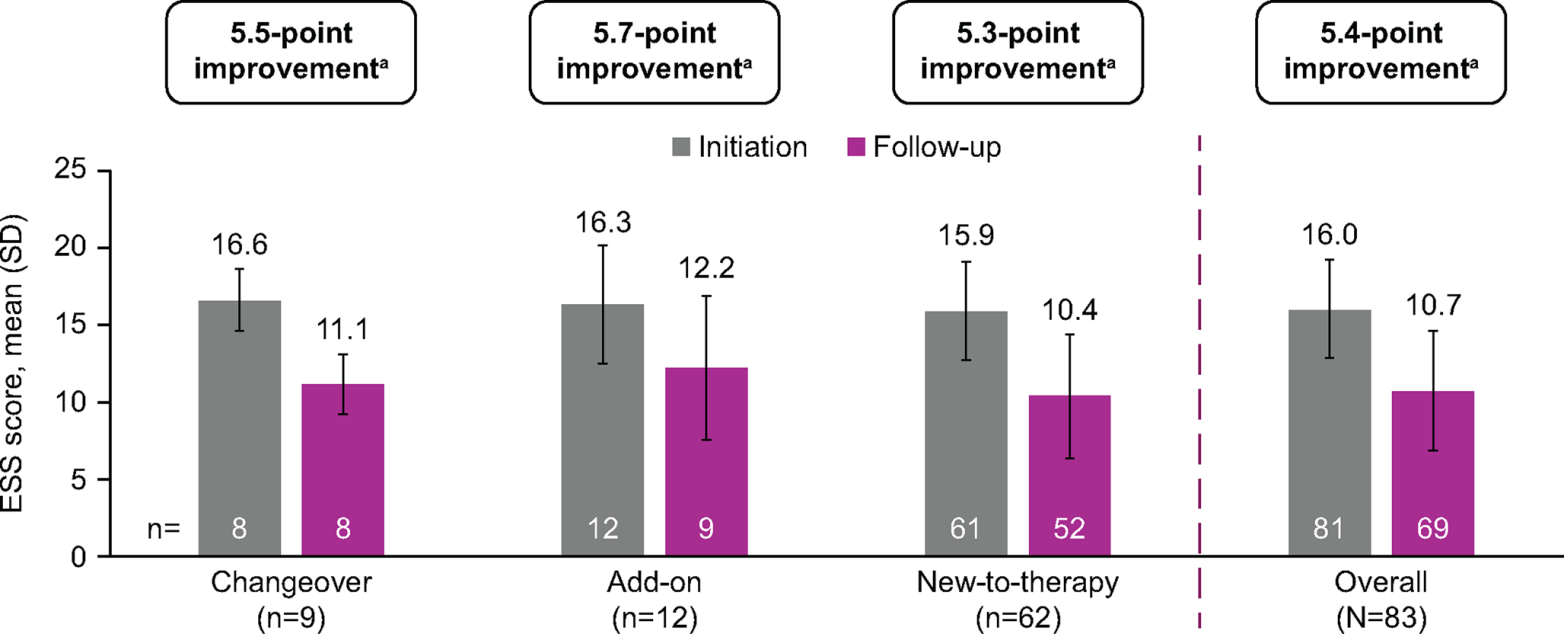
Mean Decreases in ESS Scores With Solriamfetol

The overall mean (SD) ESS score was 16.0 (3.2) at solriamfetol initiation, which decreased to 10.7 (3.9) at follow-up (mean [SD] decrease from baseline of 5.4 [3.6, *p* <.001]) (Fig. 2). Changes in ESS scores generally were consistent across subgroups, with mean (SD) decreases from solriamfetol initiation to final follow-up of 5.5 (2.9, *p* =.001) in the changeover group, 5.7 (4.8, *p* =.008) in the add-on group, and 5.3 (3.6, *p* <.001) in the new-to-therapy group.

### Patient- and physician-reported change in symptoms

**Fig. 3:**
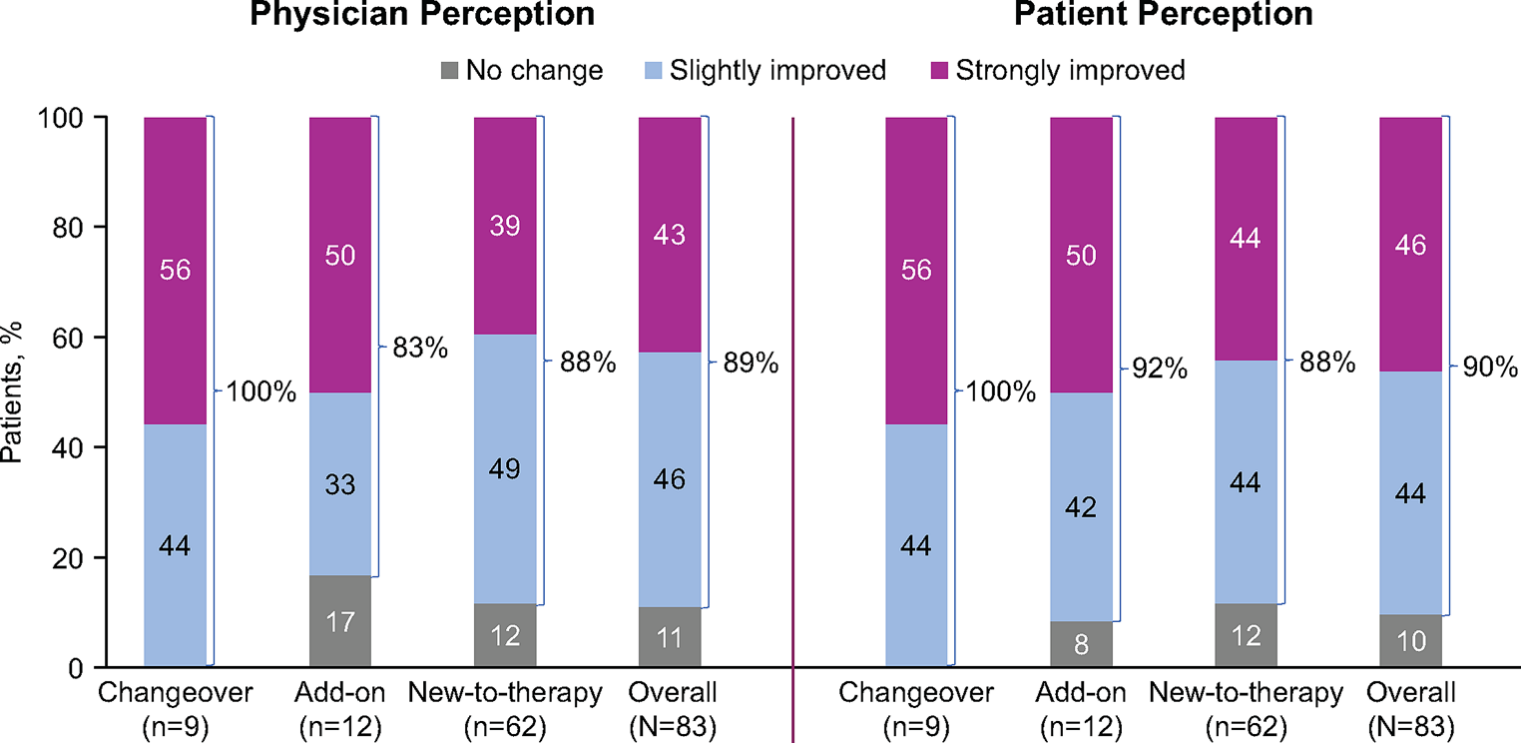
Physician and Patient Perceptions^a^ of Improvement in EDS

Most patients (*n* = 74, 90%) reported strong (*n* = 38, 46%) or slight (*n* = 36, 44%) improvement in EDS-related symptoms from solriamfetol initiation to final follow-up; this finding generally was consistent across the subgroups (Fig. 3). Similarly, physicians reported strong or slight improvement in EDS for 89% (*n* = 73) of patients (Fig. 3). Patients and physicians reported no improvement for the remaining patients (*n* = 8 and *n* = 9, respectively; data unavailable for 1 patient); there were no patient or physician ratings of slight or strong worsening of symptoms.

### Patient-reported duration of effects

**Fig. 4:**
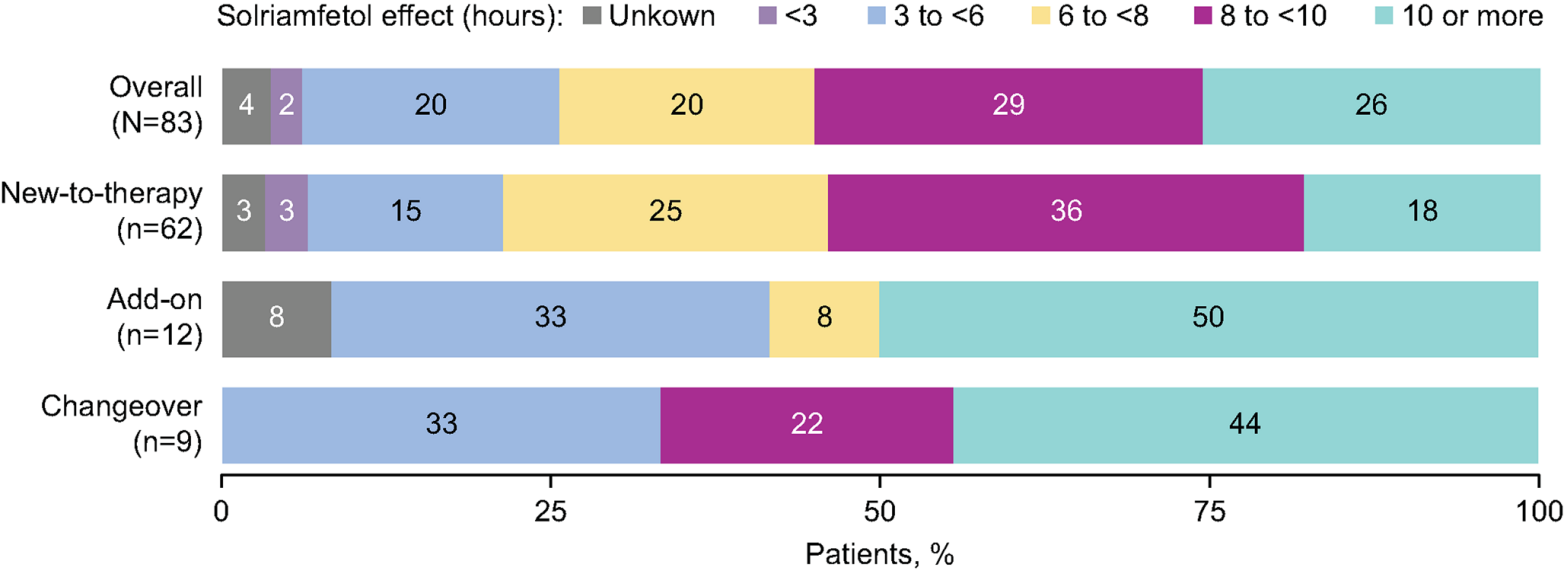
Patient-Reported Duration of Effects of Solriamfetol

Most patients overall (*n* = 45, 55%) and by initiation strategy (changeover, *n* = 6, 67%; add-on, *n* = 6, 50%; new-to-therapy, *n* = 33, 54%) reported that the effects of solriamfetol lasted ≥ 8 h. Across initiation strategies, 24 patients (29%) reported they perceived the effects of solriamfetol lasting 8 to < 10 h, while 21 (26%) reported effects lasting ≥ 10 h (Fig. 4).

Overall, patients were not equally likely to endorse no wearing off, gradual wearing off or abrupt wearing off (*p* <.001). The majority (50%, *n* = 41) of patients reported no wearing off of solriamfetol at the end of the day, and 41% (*n* = 34) reported a gradual decrease of the therapeutic effects of solriamfetol; only 4% (*n* = 3) reported abrupt wearing off. Responses were unknown for 5% (*n* = 4) of patients. Findings generally were consistent across initiation strategies.

### Patient-reported nighttime sleep quality

Overall, 75 patients (91%) reported their nighttime sleep quality did not meaningfully change after solriamfetol initiation (changeover, *n* = 8, 89%; add-on, *n* = 10, 83%; new-to-therapy, *n* = 57, 93%); however, some patients reported that sleep improved (changeover, *n* = 1, 11%; add-on, *n* = 1, 8%) or worsened (new-to-therapy, *n* = 3, 5%).

### Adverse events

A total of 27 patients (33%) reported an adverse event. The most frequent adverse events were headache (9%), decreased appetite (7%), and insomnia (6%). Headache and decreased appetite were reported across initiation strategies, whereas insomnia was reported only in the new-to-therapy subgroup (Table 2). Increased blood pressure (*n* = 1, 2%) and increased heart rate (*n* = 1, 2%) were reported in the new-to-therapy subgroup.

**Table 2:** Treatment-emergent adverse Events^a^

TEAE, *n* (%)	Changeover (*n* = 9)	Add-On (*n* = 12)	New-to-Therapy (*n* = 62)^b^	Overall (*N* = 83)^b^
Any TEAE	2 (22)	7 (58)	18 (30)	27 (33)
Headache	1 (11)	1 (8)	5 (8)	7 (9)
Decreased appetite	1 (11)	1 (8)	4 (7)	6 (7)
Insomnia	0	0	5 (8)	5 (6)
Feeling jittery	0	2 (17)	2 (3)	4 (5)
Irritability	0	1 (8)	3 (5)	4 (5)
Dizziness	0	2 (17)	1 (2)	3 (4)
Abdominal pain	0	0	2 (3)	2 (2)
Other	0	1 (8)	2 (3)	3 (4)

^a^Reported in ≥ 2 patients

^b^TEAE information was missing for one patient

TEAE, treatment-emergent adverse event

## Discussion

Germany’s SURWEY data provide real-world insight into solriamfetol initiation and titration strategies, patient- and physician-reported changes in EDS, and adverse events among a cohort of patients with OSA. The demographic characteristics of this cohort were consistent with those of larger populations of patients with OSA. Common comorbidities such as depression and hypertension generally were also characteristic of populations of adults with OSA and EDS [8].

Most patients initiating solriamfetol were new to the EDS therapy. In alignment with recommended dosages of solriamfetol [12] and previous real-world data in patients with OSA [16], 37.5 mg/day and 75 mg/day were the dosages most prescribed at initiation; 65% of patients had their doses titrated, and most titrations were completed within 2 weeks of initiation. In decisions to initiate solriamfetol, comorbidities were the most considered factor, followed by prior and concomitant medications. Lack of efficacy of prior medication was the reported reason for changing over to solriamfetol, and switching was managed by abrupt discontinuation of other medications at initiation.

At baseline, patients in this cohort generally had moderate to severe EDS (ESS score ≥ 16 [18]), which was consistent across subgroups. In a post-hoc analysis, mean improvement in ESS score was consistent with those seen in a clinical trial following 12 weeks of treatment with solriamfetol in patients with EDS and OSA; by week 12, mean ESS scores decreased from baseline by 5.0, 5.1, and 7.7 in patients who received solriamfetol 37.5 mg, 75 mg, and 150 mg, respectively [14]. In the present study, ESS scores improved by > 5 points across all initiation strategies; improvements were notably greater than the minimum clinically important difference of 2 to 3 points [19]. It is noteworthy that most patients in the present study used doses lower than 150 mg, suggesting that changes in ESS scores were similar to those reported in clinical trials. Consistent with these findings, patient- and physician-reported changes in perceptions of EDS symptoms reflected slight or strong improvement in 90% and 89% of patients, respectively. These findings are similar to clinical data in patients with EDS and OSA, such that 12 weeks of solriamfetol led to a categorical improvement in Patient and Clinical Global Impression of Change up to 90% of patients [14]. Published SURWEY findings for patients with narcolepsy showed somewhat greater variability across initiation strategies and ESS score improvements (mean decreases, 3.7–6.1); much as in the present OSA cohort, however, the narcolepsy cohort’s patient- and physician-reported perceptions reflected slight or strong improvement in ≥ 90% of patients [17].

In the present study, most patients reported perceiving the effects of solriamfetol lasting ≥ 8 h, with many patients reporting ≥ 10 h of effect. All add-on patients who perceived ≥ 8 h of effects reported they lasted ≥ 10 h. One explanation for this may be an additive effect of solriamfetol when administered with other medications to treat EDS. In this context, 58% and 17% of patients in the add-on therapy group were also prescribed pitolisant and modafinil, respectively. Previous clinical trial data for patients with OSA demonstrated that the effects of solriamfetol dosed at ≥ 75 mg/day were maintained through 9 h [14]. Consistent with previous research [12], most patients reported no perceived impact of solriamfetol on nighttime sleep quality. While some patients reported insomnia as an adverse event, such reports were specific to patients new to therapy.

In the present study, common adverse events included headache, decreased appetite, and insomnia, which are consistent with the known safety profile of solriamfetol [14, 15]. The incidences of headache and decreased appetite generally were consistent across subgroups, and these adverse events were the only ones reported by patients who changed over from a different medication. Changes in blood pressure during treatment, as measured by doctors, were uncommon. One patient reported increased blood pressure, which was physician confirmed and did not exceed 35% of the baseline value, and 1 patient reported increased heart rate; both patients were new to therapy.

Real-world data on solriamfetol in patients with OSA and associated EDS are limited. A chart review study of US-based patients and physicians—the Solriamfetol Titration & AdministRaTion (START) study [16]—yielded results similar to those of the present study. START did not evaluate treatment outcomes, but, much as in this SURWEY cohort, most of its patients underwent titration, and most who switched to solriamfetol did so because of lack of efficacy of prior medications. In addition, 37.5 mg/day and 75 mg/day were the most common starting doses for patients with OSA in both studies, although more patients overall in START initiated solriamfetol at 75 mg/day. In contrast to the present study, most new-to-therapy patients in START initiated solriamfetol at 75 mg/day, while most transitioning from a different medication started at 37.5 mg/day. Differences in prescribing practices in US and German physicians might explain the difference in initiation doses between START and the present study.

SURWEY included data from European physicians experienced in treating EDS associated with OSA, highlighting the strength of these results in the context of real-world data in this patient population. As such, these results expand on previous real-world data [16] and broaden the scope of available data on the use of solriamfetol to treat EDS. In particular, the inclusion of records from patients diagnosed with comorbid psychiatric conditions broadens the generalizability of these findings beyond those from clinical trials, which have more restricted populations [20]. Nevertheless, the present study had several limitations. First, variability in responses to the survey questions made it difficult to interpret the data on titration schedules and each patient’s final and stable doses, thus these data are not presented. In addition, the study did not include formal statistical analyses, and, as such, all results are presented descriptively or with post-hoc statistical analyses. Further, because only patients who had completed ≥ 6 weeks of solriamfetol treatment were included, results for patients unable to remain on treatment were not evaluated. Given these constraints, the present findings do not provide comparative insight into short-term versus long-term treatment outcomes.

## Conclusions

This study provides medium-term real-world data on the use of solriamfetol in patients with OSA and associated EDS in Germany. The majority of patients were new to therapy. Solriamfetol was initiated at 37.5 mg/day in most add-on and new-to-therapy patients, compared with 75 mg/day in most changeover patients; titration after initiation was common and in most patients was completed within 2 weeks. Improvement in EDS was notable and consistent with clinical trials, with an overall average ESS score decrease of 5.4, from baseline to final follow-up, and with patient- and physician-reported improvement in EDS symptoms in ~ 90% of patients. Common adverse events were consistent with those reported in patients with OSA and EDS treated with solriamfetol in previous clinical trials.

## Data Availability

All relevant data are provided within the manuscript and supporting files.
