# Correction to: SURWEY real-world study of solriamfetol: initiation, titration, safety, efficacy, and follow-up experience for patients with obstructive sleep apnea in Germany

**DOI:** 10.1007/s11325-025-03321-3

**Published:** 2025-04-09

**Authors:** Yaroslav Winter, Geert Mayer, Heike Benes, Lothar Burghaus, Samantha Floam, Gregory S. Parks, Ulf Kallweit

**Affiliations:** 1 Department of Neurology, University of Saarland, Homburg, Germany; 2 Department of Neurology, Philipps University Marburg, Marburg, Germany; 3 Hephata Klinik, Schwalmstadt, Germany; 4 Somni bene GmbH Institut für Medizinische Forschung und Schlafmedizin Schwerin GmbH, Schwerin, Germany; 5 Department of Neurology, Heilig Geist-Hospital, Cologne, Germany; 6 Axsome Therapeutics, New York, USA; 7 Formely of Axsome Therapeutics, New York, USA; 8 Center for Biomedical Education and Research, and Professorship for Narcolepsy and Hypersomnolence Research, Department of Medicine, University Witten/Herdecke, Witten, Germany; 9 Department of Neurology, University of Saarland, Kirrberger Str, 66421 Homburg, Germany

**Correction to: Sleep and Breathing (2025) 29:112**

10.1007/s11325-025-03275-6

In the originally published version of this article, the article title was incorrectly given as “Patients with obstructive sleep apnea in Germany.” This has been corrected to read “SURWEY real-world study of solriamfetol: initiation, titration, safety, efficacy, and follow-up experience for patients with obstructive sleep apnea in Germany.”

The original article has been corrected.

